# Effectiveness of an Interactive Web-Based Clinical Practice Monitoring System on Enhancing Motivation in Clinical Learning Among Undergraduate Nursing Students: Longitudinal Quasi-Experimental Study in Tanzania

**DOI:** 10.2196/45912

**Published:** 2025-04-23

**Authors:** Patricia Herman, Stephen M Kibusi, Walter C Millanzi

**Affiliations:** 1 Department of Nursing College of Health and Allied Sciences Ruaha Catholic University Iringa United Republic of Tanzania; 2 Department of Public Health and Community Health Nursing School of Nursing and Public Health The University of Dodoma Dodoma United Republic of Tanzania; 3 Department of Nursing Management and Education School of Nursing and Public Health The University of Dodoma Dodoma United Republic of Tanzania

**Keywords:** clinical monitoring system, clinical practice, motivation in clinical learning, nursing students, smartphone, mobile phone, Ruaha Catholic University, web-based teaching

## Abstract

**Background:**

Nursing students’ motivation in clinical learning is very important not only for their academic and professional achievement but also for making timely, informed, and appropriate decisions in providing quality and cost-effective care to people. However, the increased number of students and the scarcity of medical supplies, equipment, and patients, just to mention a few, have posed a challenge to educators in identifying and navigating the best approaches to motivate nursing students to learn during their clinical placements.

**Objective:**

This study primarily used descriptive and analytical methods to examine undergraduate nursing students’ desire for clinical learning both before and after participating in the program.

**Methods:**

An uncontrolled longitudinal quasi-experimental study in a quantitative research approach was conducted from February to March 2021 among 589 undergraduate nursing students in Tanzania. Following a baseline evaluation, nursing students were enrolled in an interactive web-based clinical practice monitoring system by their program, institution, names, registration numbers, and emails via unique codes created by the lead investigator and trainers. The system recorded and generated feedback on attendance, clinical placement unit, selected or performed clinical nursing procedures, and in-between and end-of-shift feedback. The linear regression was used to assess the effect of the intervention (interactive web-based clinical practice monitoring system) controlled for other correlated factors on motivation in clinical learning (outcome) among nursing students. Nursing students’ sociodemographic characteristics and levels of motivation in clinical learning were analyzed descriptively while a 2-tailed paired sample *t* test established a comparative mean difference in motivation in clinical learning between the pretest and the posttest. The association between variables was determined using regression analysis set at a 95% CI and 5% statistical significance.

**Results:**

The mean age of study participants (N=589) was 23 (SD 2.69) years of which 383 (65.0%) were male. The estimated effect (β) of a 3-week intervention to improve nursing students’ motivation in clinical learning was 3.041 (*P*=.03, 95% CI 1.022-7.732) when controlled for other co-related factors. The mean score for motivation in clinical learning increased significantly from the baseline (mean 9.31, SD 2.315) to the postintervention (mean 20.87, SD 5.504), and this improvement presented a large effect size of 2.743 (*P*<.001, 95% CI 1.011-4.107).

**Conclusions:**

Findings suggest that an interactive web-based clinical practice monitoring system is viable and has the potential to improve undergraduate nursing students’ motivation for clinical learning. One alternative clinical pedagogy that educators in nursing education can use to facilitate clinical learning activities and develop motivated undergraduate nursing students is the integration of such technology throughout nursing curricula.

## Introduction

Owing to changes in population, technology, and communicable and noncommunicable diseases, the uncertain nature of health care provision necessitates improvements in health care systems and the teaching and learning environment across health disciplines, including nursing [1]. Clinical nursing education, in particular, has become a fundamental aspect of the nursing profession that informs educators about the best and most innovative pedagogical strategies that are navigated into technology to increase nursing students’ motivation in clinical learning and thus ensure consistent clinical attendance and passionate clinical learning around the world [2]. In clinical learning, motivation refers to an individual’s inner drive toward an activity or behavior that defines his or her achievements, in this case, the attainment of skills and competence required in the nursing profession among nursing students [3]. Scholars have shown that motivated students can demonstrate metacognition, metacompetencies, and a sense of independence when providing high-quality, cost-effective health care to a diverse population [4-6].

Educators in nursing education are important individuals in both classroom and clinical teaching and learning activities to enhance nursing students’ motivation in clinical learning during clinical placements for meta-competencies in diagnosing and making informed and appropriate decisions in providing quality and cost-effective care to people. Educators in nursing education are expected to be developed and empowered with pedagogical competencies to cope with advanced science and technology, increased rates of nursing student enrollments in middle and higher education institutions, scarcity of medical supplies and equipment, unimproved clinical teaching and learning environments, and availability of clients or patients as important individuals in clinical nursing education [7,8]. Empowering educators with knowledge and skills for facilitating clinical teaching and learning activities for nursing students may be intimately related to their pedagogical competencies in creating, supporting, mentoring, coaching, supervising, monitoring, and assessing nursing students [9,10].

Nursing students, developed by competent educators in nursing education, are believed to share their learning experiences with both educators and peers. They show motivation in their clinical learning activities and client or patient care provision as they demonstrate a sense of independence, a positive professional identity, and the attitude, skills, and values of lifelong learners and professionals [11]. Motivating nursing students to learn has risen to be prominent in clinical nursing education because it is regarded as a critical component in promoting consistent clinical attendance and learning [12]. Investing in the motivation of nursing students also ensures the development of competent nursing graduates who can demonstrate safe, ethical, and legal practices, which are foundational and essential aspects of clinical nursing education [6,13]. Contrary to what is expected of educators in nursing education in the twenty-first century, their current clinical nursing education practices are more traditional, with pedagogics such as bedside tutorials, lectures, demonstrations, discussions, case studies, portfolios, and nursing meetings, to name a few, being widely used [11,14,15]. However, due to the increased enrollment rate of nursing students with the unchanging pedagogical trend, a shortage of academic faculty, and a limited number of trained clinical instructors, the aforementioned clinical pedagogics are doubted to be able to enhance interactive communication between educators in nursing education and students.

Nevertheless, they are doubted on their abilities to establish teaching and learning feedback or experiences from students and nursing students’ motivation in clinical learning [16]. Moreover, they demonstrate weaknesses in not developing them with clinical meta-competencies for providing quality and cost-effective care to people [17]. Scholars have linked the permanent implementation of conventional clinical nursing education pedagogics to a lack of interactive communication, teaching and learning feedback from students, and clinical absenteeism as remarkable signs of unmotivated nursing students worldwide [18]. The work by Rahman et al [12] has demonstrated that clinical absenteeism has been linked to an unsatisfactory clinical learning environment as well as a shortage of competent educators in nursing education. Nevertheless, the implementation of conventional clinical supervision, mentorships, support, monitoring, and evaluation measures such as registration books and follow-up books fail to manage a large group of nursing students in clinical settings [19]. Nursing students’ avoidance and lack of enthusiasm in attending their daily practical activities during their clinical placements result in substandard care delivered to clients or patients alongside unethical professional conduct and poor customer care [20].

This work is based on the belief that understanding and implementing novel clinical pedagogical strategies will assist educators in nursing education in grasping and navigating the best ways to inspire unmotivated students to learn in a clinical context over conventional pedagogies. Adoption and integration of technology have been prioritized and have proven to be timely, quick, cost-efficient, long-term, and beneficial in enhancing students’ motivation in their learning activities [21,22]. Authors of this work agree with other scholars that it appears to be timely for clinical nursing education to transform its pedagogics to technology-based ones to fill educational gaps demonstrated by conventional pedagogics for enhancing nursing students’ motivation in clinical learning, particularly in low- and middle-income countries such as Tanzania [23]. As it has worked elsewhere, web-based learning is currently becoming an increasingly vital instructional tool in nursing education, as it offers the potential to promote motivation to learn among students [24].

Scholars including Mico et al [25] have highlighted that the web-based learning approach, which has been endorsed as an essential educational tool, responds effectively and efficiently to nurses’ needs and experiences in their clinical practices. Web-based technology in education is linked with a networking interactive model to promote communication with a feedback mechanism to enhance active learning between the users [26]. However, little about the integration of technology in clinical nursing education has been documented in Tanzania to mentor, supervise, support, monitor, and evaluate nursing students during their clinical placement [27]. If the situation remains unattended, nursing students will continue to be developed conventionally and continue to be unmotivated in their clinical learning, gaining little clinical competencies to work independently and confidently to deliver ethical, quality, and cost-effective care to people.

Therefore, this study intended to fill the gap by primarily using descriptive and analytical methods to examine undergraduate nursing students’ desire for clinical learning both before and after participating in the program.

## Methods

This study was conducted by taking into consideration international and national research standards and ethics. Moreover, it was informed by the institutional postgraduate guidelines and regulations of The University of Dodoma [28].

### Study Location

The study was conducted in Dodoma Regional Referral Teaching Hospital in Tanzania, which accommodates a large number of nursing students from different nursing training institutions within Dodoma region. Some previous scholars [11,29,30] suggest that proximity to the research environment where the intervention is being piloted or implemented aids in receiving rapid input from the consulted experts, trainers, and participants, and ensuring periodic monitoring of its integrity. Aside from the availability of nursing students, the location was chosen because of the availability and accessibility of the consulted experts and trainers for their evaluation and appraisal of the web-based clinical monitoring system throughout the design and piloting procedures.

### Study Design and Approach

As it has also been used by some previous scholarly works [31], this study used an uncontrolled longitudinal quasi-experimental design (pre-post tests) with a quantitative research approach among 589 randomly selected undergraduate nursing students in Tanzania from February 2021 to March 2021. The study began with a screening method to choose individuals who satisfied the inclusion criteria and were willing to engage in the study, followed by a baseline assessment to determine their initial level of motivation in clinical learning. Following the intervention, the same participants were exposed to the system for 3 weeks, with 1 week set apart for the end line evaluation (posttest) after the intervention as a follow-up assessment.

### Study Population

To maximize the diversities of an intervention’s effects, the study recruited undergraduate nursing students in diploma and bachelor’s degree programs from 2 middle and 2 higher training institutions in the Dodoma region of Tanzania’s central region.

### Sample Size Determination

The minimum sample size of this study was determined based on the findings from previous studies [5]. Their findings revealed a baseline mean score of knowledge about how to plan learning activities of 53.10, whereas the end line mean score was 54.21. The following formula was used to determine the minimum sample size as suggested by previous studies [29,31] to be used when researchers wish to conduct an uncontrolled quasi-experimental study design:



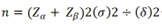



where n=minimum sample size

*Zα*: Tabulated *Z* value set at 95% (1.96) CI

*Zβ*: Tabulated *Z* value set at 80% (0.84) power to demonstrate a statistical difference between pretest and posttest.

*σ*: Polled SD = 7.511399669835177

SD_1_: SD 1 (from previous studies = 10.21)

SD_2_: SD 2 (from previous = 11.02)

*δ*: Mean difference (*M*_2_ – *M*_1_)^2^ = 0.11

*M*_1_: Mean 1 (from previous studies = 53.10)

*M*_2_: Mean 2 (from previous studies = 53.21)

With the addition of a 10% attrition adjustment of the calculated sample size (n=54) = (535+54) = 589. Therefore, the minimum sample size of this study was 589 nursing students.

### Recruitment Procedures of the Study Participants

**Figure 1:**
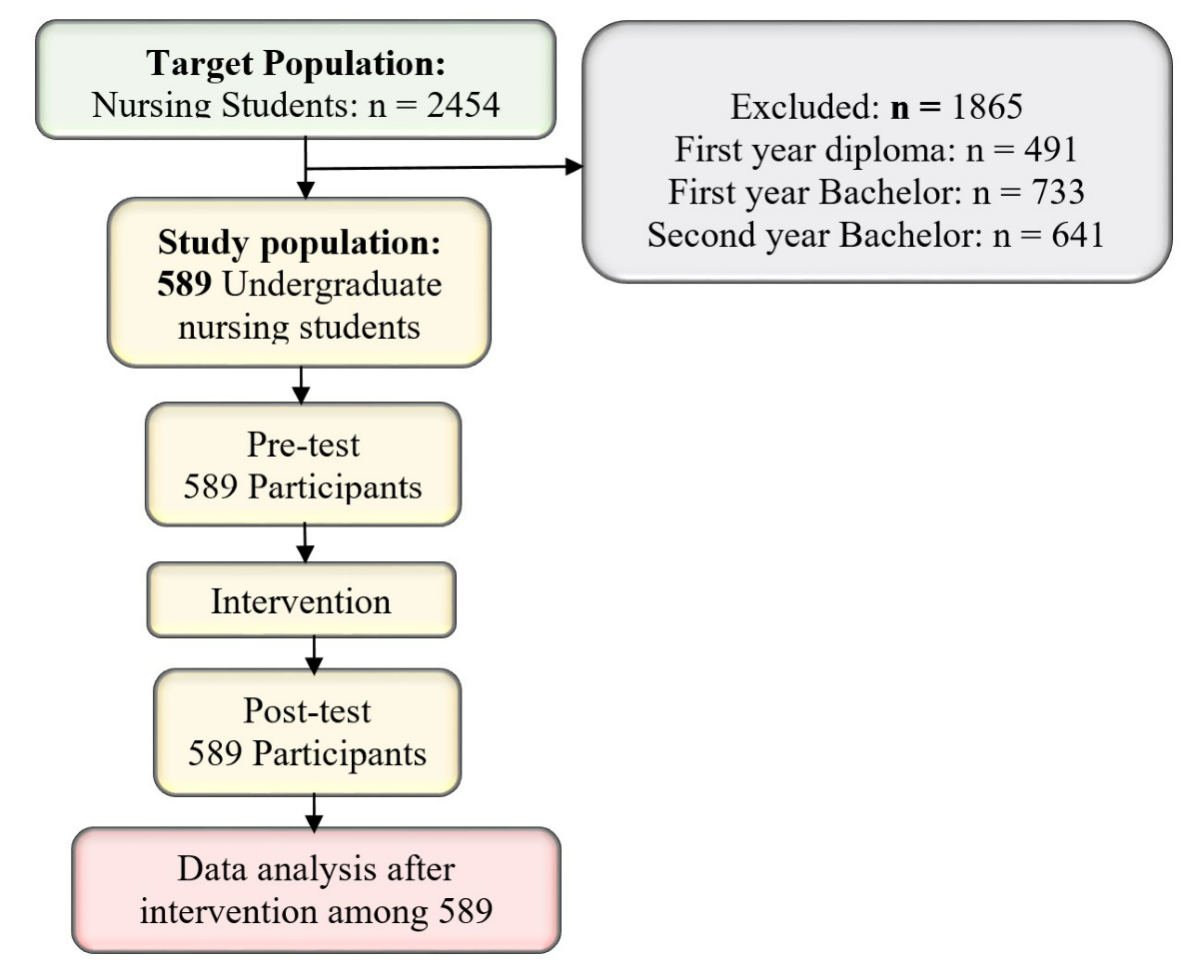
A flow pattern of sampling procedure among nursing students. From field data (2021).

The framework of recruiting study participants has been benchmarked from some previous scholarly works [11,32-37]. As shown in Figure 1, 2454 nursing students were eligible to join the study. However, 589 nursing students met the inclusion criteria and participated in the study, assessed at baseline, at end line, and their data analyzed. Sums (1865/2454, 76%) were excluded due to various reasons including not having started clinical placements (first-year diploma in nursing [n=491], a first-year bachelor of science in nursing [n=733], and a second-year bachelor of science in nursing [n=641]). There was no loss to follow-up among nursing students who joined the study and thus the completion rate was 100%.

### Eligibility Criteria

#### Inclusion Criteria

Nursing students were recruited for this study based on their willingness after being informed about the purpose, benefits, and drawbacks of participating in this study. The study recruited second-, third-, and fourth-year undergraduate nursing students who were in clinical placements at the time of this study. In addition, undergraduate nursing students with Information and Communication Technology literacy and those with iPhones or iPads were recruited for the study.

#### Exclusion Criteria

Nursing students who reported being unwell and unable to converse or participate in the study were excluded from the study. Students without institutional registration numbers, those unable to use computers or smartphones, and nursing students recruited for other studies or projects were not eligible to participate in this study.

### Sampling Procedures

As recommended by previous scholars [38-41], probability sampling techniques through multistage sampling methods were used to reach and study nursing students in this study. Stage 1: a simple random sampling technique by lottery method was used to select regions and districts. Stage 2: As shown in Tables 1 and 2, stratified sampling methods were used to select higher training institutions (institutions A and B). On the other hand, lower training institutions (institutions C and D) were randomly selected using a stratified sampling technique.

Nevertheless, a stratified random sampling technique was used to select the nursing program (diploma in nursing and bachelor of science in nursing) and classes (who are in their clinical placement). The training health facility was selected purposely because it is only a major regional referral hospital in the Dodoma region used by multiple training institutions for training nursing students in clinical settings. Stage 3: systematic sampling methods were used to select the study participants. As shown in Tables 1 and 2, a proportionate formula: n=[P_i_×(n/p)] was used to determine a minimum sample size of nursing students per training institution and their program, respectively, whereas *n*_i_ is the proportional sample size, *P_i_* is the total targeted population, *n* is the number of nursing students, and *P* is the minimum sample size of the study.

**Table 1:** Proportionate sample size by the sampled training institutions^a^.

Name of training institution	Total population, n	Proportionate, n
Institution A	968	232
Institution B	998	239
Institution C	367	88
Institution D	121	30

^a^From Study plan (2021). Total number of nursing students among the sampled training institutions = 2454 (*P*_i_ × *n*/*P*).

**Table 2:** Proportionate sample size by training nursing program^a^.

Training institution	Nursing program	Total population, n	Proportionate (*P*_*i*_ × *n*/*P*)
Institution A	Diploma in nursing	230	55
Institution A	Bachelor in nursing	738	177
Institution B	Bachelor in nursing	828	198
Institution B	Diploma in nursing	171	41
Institution C	Diploma in nursing	367	88
Institution D	Diploma in nursing	121	30

^a^From Study plan (2021).

### Intervention

**Figure 2:**
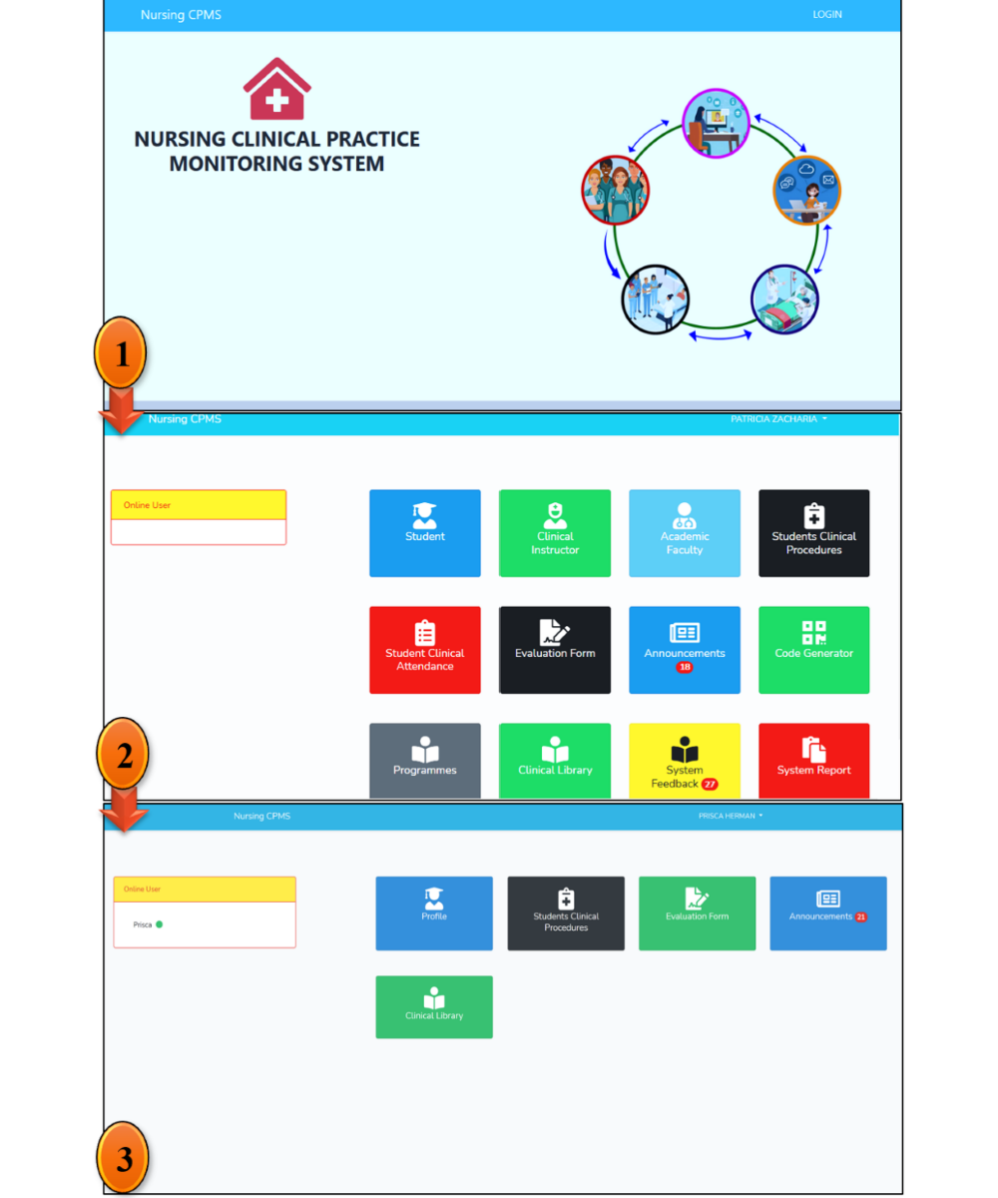
A welcoming window, menu window, and nursing students’ node with various menus in the system. From field data (2021).

The intervention was completed in 3 weeks, with 1 week set aside for the final evaluation (posttest). It was carried out on a daily basis (6 hours a day), per the clinical rotation and placement schedules established by the nursing students’ respective training institutions. Within the 3 weeks of the intervention, all students were expected to perform and complete the assigned clinical learning task or activities and models on a daily basis and within 6 hours of their duty shifts. The primary aim of the intervention was to descriptively and analytically measure a change in motivation in clinical learning among undergraduate nursing students before and after participating in the program. Trained research trainers who also had clinical nursing education competence implemented the intervention. As illustrated in Figure 2, the system included a welcoming window as well as several menus or nodes such as clinical instructors, academic staff, clinical nursing procedures, clinical attendance, evaluation forms, announcements, code generator, professional programs, clinical library, system feedbacks and reports, and students’ node, which included students’ profiles, clinical notes, attendance, evaluation forms, and announcement menus.

**Figure 3:**
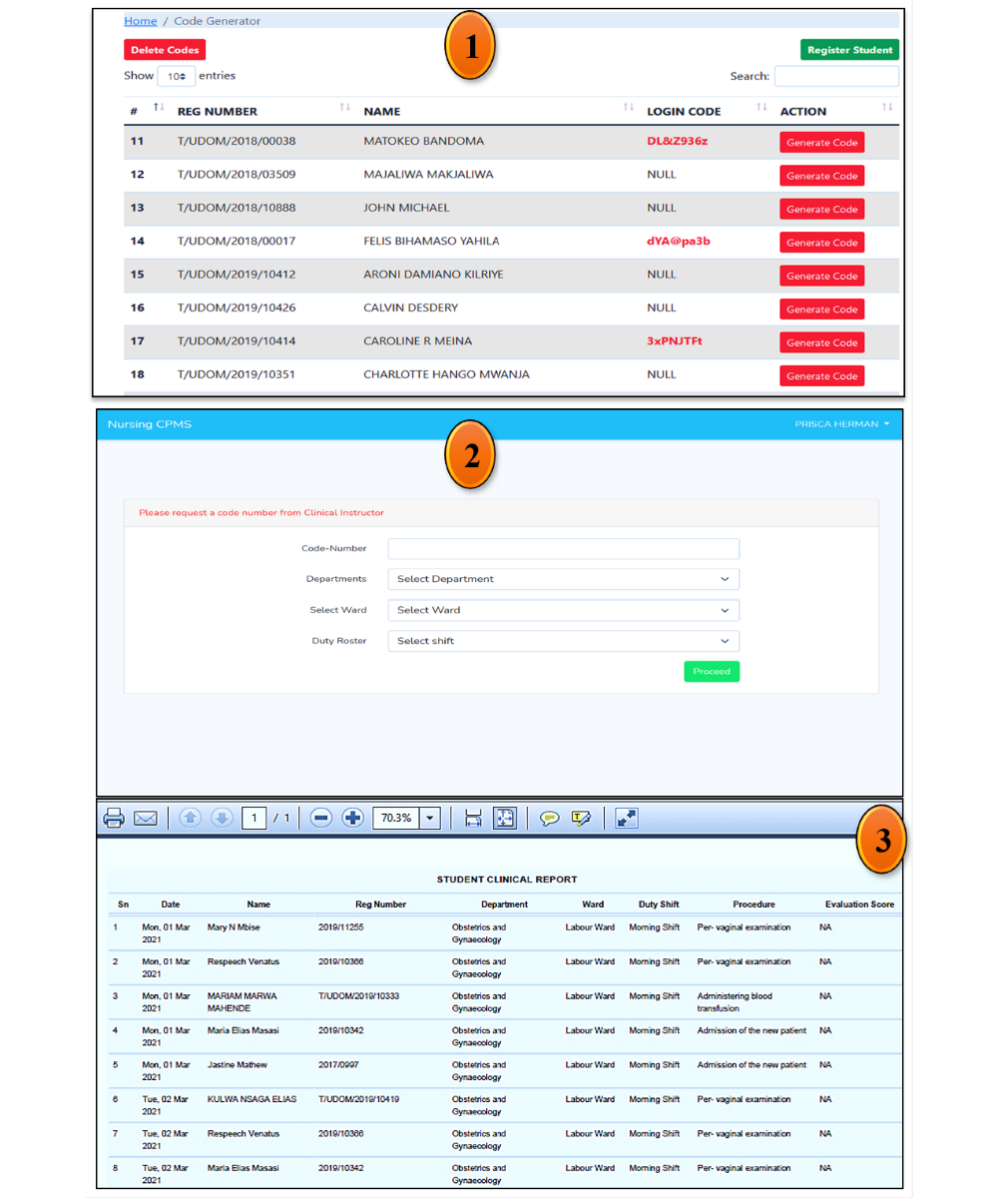
Code generator window, nursing students’ login window, and an example of a system-generated report. From field data (2021).

Referring to Figure 3, nursing students were required to arrive at their shift very early each day before the tuned time in the system, which was 7:30 AM (East African time), to be assigned codes of a specific day or date generated by the trainers. The generated codes allowed them to log in to the system and sign out in the presence of the trainer at the end of the shift, which was tuned at 1:30 PM (East African time). Any student who arrived late for his or her shift and who did not provide web-based feedback on his or her performance of the chosen clinical nursing procedure in the presence of clinical instructors or trainers in between shifts, or left the shift before the fixed time of signing out was considered absent in that particular shift. The system provided a daily shift attendance report that included the dates, students’ names and registration numbers, department, ward or unit, duty shift, clinical nursing procedure executed, and evaluation score for each student.

**Figure 4:**
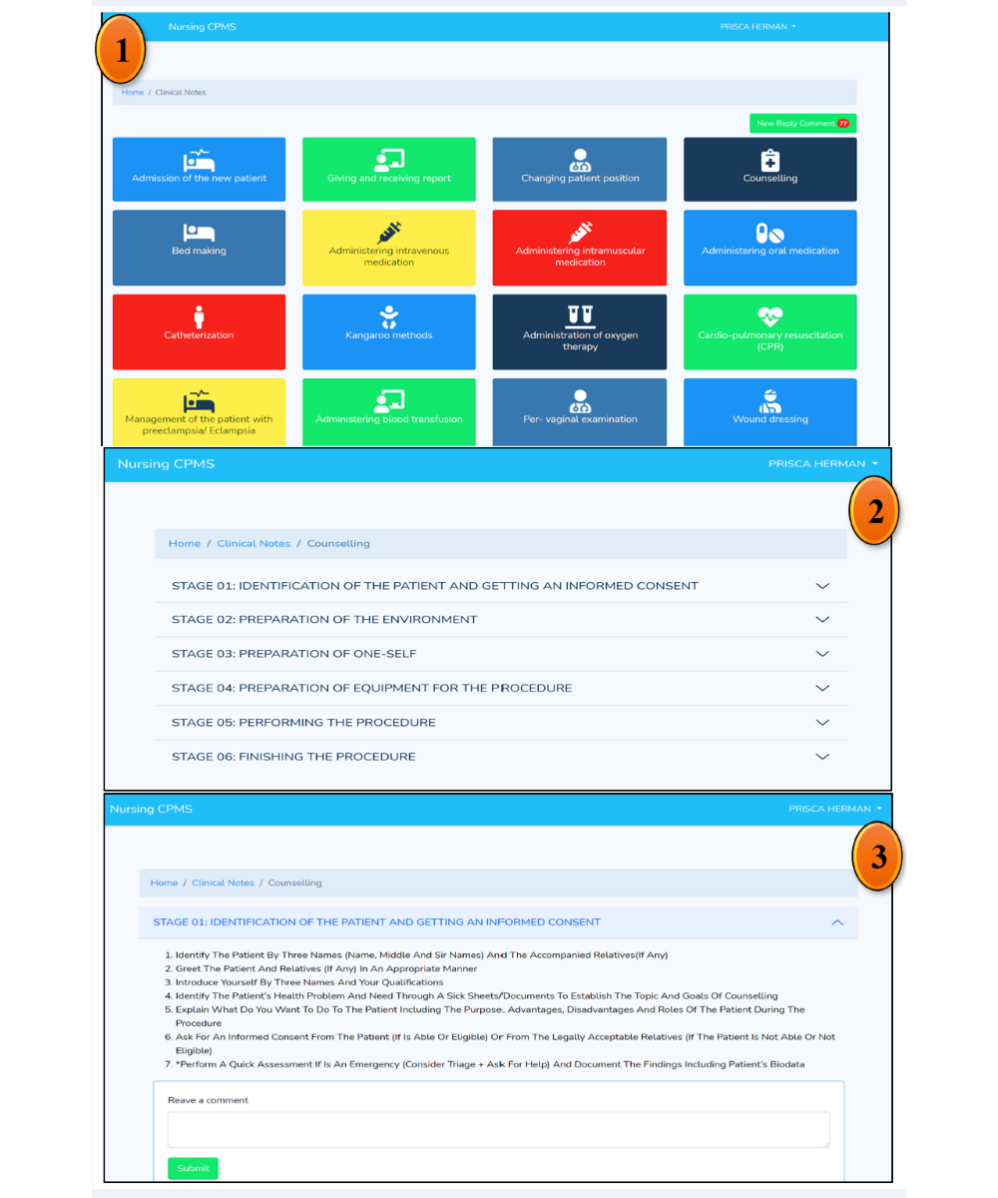
Variety of clinical nursing procedures, stages, and activities imported into the system for nursing students. From field data (2021).

Nonetheless, as shown in Figure 4, several nursing clinical procedures were imported into the system, including giving and receiving reports; patients admission; changing patients’ positions on a bed; counseling; bed making; administration of intravenous, intermuscular, and oral medications; catheterization; kangaroo mother care; administration of oxygen to patients (oxygen therapy); cardiopulmonary resuscitation; management of patients with preeclampsia or eclampsia; blood transfusion; per-vagina examination; wound dressing; mouth care; management of postpartum hemorrhage; assessment of placenta; management of pregnant women in a first and second stage of labor; assessment of new-born babies; history taking during labor; and vital sign assessment. After selecting a clinical nursing procedure for a particular duty shift, nursing students had to adhere to the 6 stages of conducting it including identification of the patient alongside getting informed consent; preparation of the environment; one-self; equipment appropriate for the chosen procedure; and performing and finishing the procedure. Each clinical nursing procedure was featured in several activities, which nursing students were supposed to follow, adhere, and implement chronologically.

### Procedures for Getting Nursing Students Into the System, Supervising, Supporting, Monitoring, and Evaluating Them

Undergraduate nursing students were recruited based on their units of clinical placements including the emergency unit, labor and postnatal ward, pediatric ward, and male medical ward within Dodoma Regional Referral Hospital. Resources such as computers, smartphones or iPads, electricity, web connectivity, clinical nursing procedure checklists, duty rosters, notebooks, pens, and shift objectives were the requirements for the intervention. Nursing students’ institutional registration numbers were used as username identities during the login procedures into the system.

Clinical nursing procedures and evaluation checklists were imported into the system during the intervention to allow nursing students and clinical instructors to access them and have an interactive room to identify and discuss them before starting to care for the patients on that particular day. Nursing students had to ask for a system-generated code in the presence of a (system implementers) clinical instructor to be able to log in to the system and counted to have reported timely in a particular duty shift as the mode of monitoring and keeping clinical attendance reports among them. Furthermore, after the successful registration of nursing students into the system, system implementers had to confirm the presence of nursing students throughout the specified duty shift.

A shift progress Min-reports (brief or summary report of what a student has done for a particular time of a shift) was posted by nursing students in the system including patients’ progress after performing the procedure. On the other hand, trained research trainers had the role of periodically posting announcements into the system to enhance interactive and reciprocal communication among nursing students and academic faculty. They also had the role of summing up nursing students’ clinical signs of progress and filling the evaluation forms at the end of a duty shift. All clinical teaching and learning activities performed by the trained research trainers and nursing students were tracked, monitored, and sometimes addressed by the principal investigator (PZH) through a system WebTop. All self-rated evaluations on the performed clinical procedures, day feedback, and experiences of the interactive web-based clinical practice monitoring system among nursing students and system implementers were captured through the system before they signed out at the end of the duty shift.

**Figure 5:**
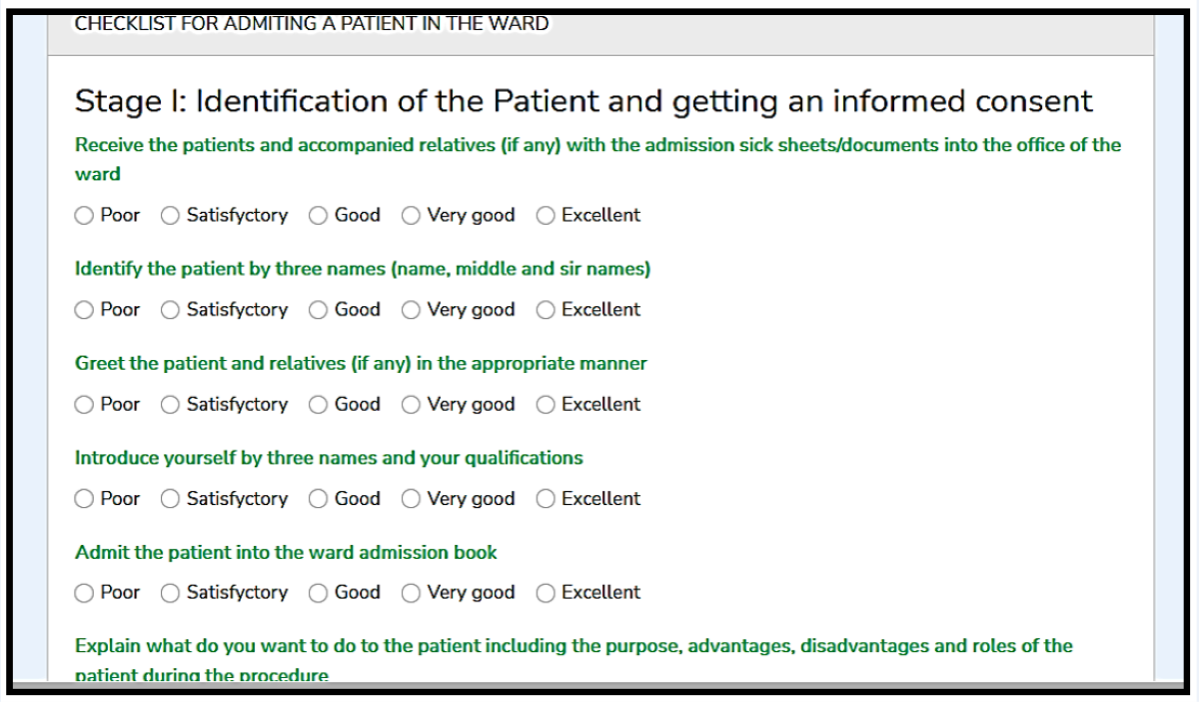
An example of a self-rated evaluation form imported into the system for nursing students. From field data (2021).

Only 1 session (morning duty shift) was preferred for the intervention as negotiated by hospital administration and system implementers. The intervention was implemented for 6 hours equivalent to 1 duty shift in a day with a duration ranging from 7:30 AM to 1:30 PM (East African time) among nursing students. Each student had to carry out and finish one of the aforementioned clinical nursing procedures every day for 3 weeks for learning to take place. This amounted to about 20 clinical nursing procedures in total. All students finished the same clinical nursing procedures during the intervention’s 3-week duration including giving and receiving reports; patients admission; changing patients’ positions on a bed; counseling; bed making; administration of intravenous, intermuscular, and oral medications; catheterization; kangaroo mother care; administration of oxygen to patients (oxygen therapy); cardiopulmonary resuscitation; management of patients with preeclampsia or eclampsia; blood transfusion; per-vagina examination; wound dressing; mouth care; management of postpartum hemorrhage; assessment of placenta; management of pregnant women in first and second stages of labor; assessment of newborn babies; history taking during labor; and vital sign assessment. Assessment of nursing students’ motivation in clinical learning was performed using academic motivation questionnaires that were administered to them as pretests and posttests. As shown in Figure 5, the evaluation of the system was performed through nursing students’ and system implementers’ experiences feedback inventory that was posted in the system and filled out before signing out of the system.

### Strategies Used to Maintain Adherence to the Intervention Among Nursing Students

This section describes the measures used to increase nursing students’ adherence during intervention implementation in the field. Throughout the intervention, nursing students’ registration numbers were used as identifiers during system deployment. Being unidentified and acknowledged by name would most likely make nursing students feel at ease and confident in their clinical learning privacy, allowing them to feel free and inspired to follow the intervention regimen. Furthermore, with the assistance of academic faculty and clinical instructors, the study was carried out under the training institutional clinical rotation schedule, which most likely aided this study in ensuring that nursing students adhered to the intervention because they had to attend their shifts accordingly. The use of technology to assist clinical learning among nursing students appeared to impress them, particularly the ways interactive communications and physical contact were assured to them. Nursing students and system implementers have enhanced adherence to the intervention among nursing students by posting various announcements, peer teaching and learning, and self-evaluation forms.

### Data Collection Procedures

The focus of the current effort was mostly on presenting the study’s quantitative findings. Quantitative data were gathered at two-time intervals, baseline, and end line (directly postintervention), in different unoccupied rooms, as agreed upon by the administrative authority of the respective training institutes. The research assistants were chosen on purpose based on their eligibility criteria, which included at least 1 year of data-gathering experience. Nursing students were given a brief explanation of completing the questionnaire before having copies provided by research assistants. The researcher and assistants were present throughout the process to supervise and address the overstretched immediate and long-term problems before collecting all copies of completed surveys and protecting them as part of nursing students’ confidentially. Before leaving the room, nursing students were informed of the timetable and method for intervention.

### Research Tools and Instruments

The data collection tool for the quantitative part of the work consisted of 2 parts: the sociodemographic characteristics profiles and the part that measured nursing students’ motivation in clinical learning. Gender, age, marital status, interest, motive for joining the nursing profession, accommodation, and marital status were all part of the sociodemographic profile. The study used a 5-point Likert scale to assess motivation in clinical learning using an Academic Motivation Scale composed of 28 items adopted from previous scholarly works [5,42-44]. The 5-point Likert scale ranged from 1=strongly disagree to 5=strongly agree. Its subscales included intrinsic motivation which was assessed by 12 items, extrinsic motivation (12 items), and amotivation (4 items) of which the findings were dichotomized into an “Agree” (assigned a value of “1”) response that described the action to have been performed by the participant; otherwise, “Disagree” (“0” value) for unperformed action or behavior.

Before data collection, the principal component analysis was performed at a measure of Keiser-Meyer-Olkin and Bartlett test value of >0.5 and a significance level of ≤5% to measure the weight of each item. The findings revealed that all 28 items scored >0.3, and thus, they were all retained for data collection. Scoring the variable of motivation in clinical learning was adopted in a study conducted by Millanzi and Kibusi [6] that nursing students who scored 0-16 were categorized into low motivation in clinical, those who scored 17-24 had moderate learning motivation, and nursing students who scored 25 and above demonstrate high motivation in clinical learning.

### Validity and Reliability of the Study

The validity and reliability tests were performed first before subjecting the tool to the actual field for data collection. The tool was shared with the subject matter and statistician for suitability of the items, reliability, and ambiguity to fit for knowledge to undergraduate nursing students. The pretest of research tools was conducted among 60 nursing students at a location that was different from the sampled study setting. The finding of a pretest was subjected to the exploratory factor analysis to determine the weight of each item to assess the outcome of interest and the reliability scale analysis to determine the internal consistency of the tool and presented using Cronbach α values as recommended by previous studies [45]. The findings of the scale analysis on motivation in clinical learning were found to be Cronbachα=0.840 (>0.7), which was statistically reliable for the actual data collection.

### Data Analysis

An SPSS software program (version 23; IBM Corp) was used to analyze data. Before data analysis, cleaning was done to ensure the completeness, accuracy, and clarity of the information in the questionnaires. A normality test was performed to determine the distribution of data to opt for the mode of data analysis. Findings of the normality test revealed that data motivation in clinical learning was approximately normally distributed, and thus, parametric statistical measurements were adopted. Descriptive statistic was used to establish participants’ sociodemographic characteristics profiles of the study participants such as age, sex, program, and year of study, just to mention a few, and the motivation in clinical learning. For inferential statistics, a 2-tailed paired *t* test was performed to determine a comparative mean score change and difference in motivation in clinical learning among nursing students between the pretest and the posttest.

The multiple linear regression analysis model by considering the control of other factors as independent variables such as sociodemographic characteristics profiles and the intervention (interactive web-based clinical practice monitoring system) was performed to establish the extent of association with the outcome variable of interest (motivation in clinical learning). The multiple linear regression was opted for because several factors were controlled during analysis and the outcome variable of interest was treated as a scale variable. The goal of this study was to determine the net effect of the intervention by taking into account and controlling for the sociodemographic characteristic profiles of nursing students, as it was hypothesized that these profiles would also have an impact on the outcome of interest (motivation in clinical learning) over the intervention. Findings of inferential statistics were presented in tabular forms by means, of SDs, *t* values, significance level (<5%), and 95% CI. The following logistic regression model was used:



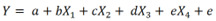



where *Y* is dependent variable; *a* is intercept; *b*, *c*, *d*, and *e* are slopes; *X*_1_, *X*_2_, *X*_3_, and *X*_4_ are independent (explanatory) variables; and *e* is residual (error).

### Ethical Considerations

This study conformed to The University of Dodoma institution’s postgraduate guidelines and regulations after being approved and given an ethical clearance (number MA.84/261/02/81) by the institutional research ethics review committee. All participants provided written informed consent after being explained about the study and their freedom to participate in it. Data collection procedures were performed in separate and unoccupied venues that were available in the respective institution premises. Participants’ names were not included in the data collection tools and their information was secured by the principal investigator (PZH) using folders with passwords. Given that participants were in their academic clinical rotations calendar for clinical learning activities, there were no compensations of either time or monetary incentives throughout the study.

## Results

### Proportional Distribution of Nursing Students by Their Sociodemographic Characteristics Profiles

The completion rate of the study was 100% of the studied participants. Findings in Table 3 indicate that 65.0% (383/589) of nursing students were males while 79.6% (469/589) of the sample were younger than 24 years, with a mean age of 23 (SD 2.689, range 19-50) years. Accommodated participants in their respective training institutions’ hostels accounted for 71.5% (421/589) while 63.7% (375/589) of them were enrolled in bachelor of science in nursing and 33.6% (198/589) and 30.1% (177/589) of them were in their fourth and third year of studies, respectively. A majority of nursing students 69.4% (406/589) were not interested in joining nursing programs. However, those who were interested in joining nursing education were driven by a belief that it is a secure profession (567/589, 96.3%), caring to save peoples’ lives (491/589, 83.4%), autonomy to practice (478/589, 81.2%), and generous salary and employment benefits (438/589, 74.4%). Other findings were found as shown in the table.

**Table 3:** Proportional distribution of nursing students by their sociodemographic characteristics (n=589)^a^.

Variable			Values					
Age (years), mean (SD; range)			23 (2.689; 19-50)					
**Age (years), n (%)**								
	<24					469 (79.6)		
	25-34					115 (19.5)		
	>35					5 (0.9)		
**Institution, n (%)**								
	Training institution A				232 (39.4)			
	Training institution B				239 (40.6)			
	Training institution C				88 (14.9)			
	Training institution D				30 (5.1)			
**Sex, n (%)**								
	Male					383 (65.0)		
	Female					206 (35.0)		
**Marital status, n (%)**								
	Single					543 (92.2)		
	Married					46 (7.8)		
**Accommodation, n (%)**								
	In-campus					421 (71.5)		
	Off-campus					168 (28.5)		
**Program of study, n (%)**								
	Diploma in nursing and midwifery					214 (36.3)		
	Bachelor of science in nursing					375 (63.7)		
**Year of study** **, n (%)**								
	Second-year diploma in nursing					89 (15.1)		
	Third-year diploma in nursing					125 (21,2)		
	Third-year bachelor of science in nursing					177 (30.1)		
	Fourth-year bachelor of science in nursing					198 (33.6)		
**Interested to join the nursing profession, n (%)**								
	No							409 (69.4)
	Yes							180 (30.6)
**Reason to join the nursing profession**								
	**Generously salary and employment benefits, n (%)**							
		Yes					438 (74.4)	
		No					151 (25.6)	
	**A secured profession, n (%)**							
		Yes		567 (96.3)				
		No		22 (3.7)				
	**Autonomy to practice, n (%)**							
		No					478 (81.2)	
		Yes					111 (18.8)	
	**Caring to save people’s lives, n (%)**							
		No		491 (83.4)				
		Yes		98 (16.6)				
	**Opportunity to travel worldwide, n (%)**							
		Yes					421 (71.5)	
		No					168 (28.5)	
	**Job availability, n (%)**							
		Yes					398 (67.5)	
		No					191 (32.5)	

^a^From field data (2021).

### The Overall Distribution of the Level of Motivation in Clinical Learning and Their Domains Among Nursing Students

The findings from Table 4 revealed that there was no significant difference between the proportion of nursing students with low and moderate motivation in clinical learning (261/589, 44.3%; and 328/589, 55.7%, respectively). However, baseline findings indicated that none of the nursing students demonstrated high motivation in clinical learning 0.0% (n=0). On the other hand, baseline findings of the motivation domains indicated that 94.7% (558/589) of nursing students were not intrinsically motivated in clinical learning contrary to the end line findings, which indicated that 90.5% (533/589) demonstrated inner motive in clinical learning. Highly motivated nursing students to learn in clinical settings accounted for 67.7% (399/589) while only 4.9% (29/589) of them demonstrated lower motivation in clinical learning. Other findings were observed as shown in Table 4.

**Table 4:** Overall distribution of the level of motivation in clinical learning and their domains among nursing students in the Dodoma region (N=589)^a^.

Variable				Pretest		Posttest
**Overall motivation in clinical learning,** **n (%)**						
	High learning motivation		0 (0)		399 (67.7)	
	Moderate learning motivation		328 (55.7)		160 (27.2)	
	Low learning motivation		261 (44.3)		29 (4.9)	
**Motivation subscales**						
	**Intrinsic motivation in clinical learning,** **n (%)**					
		No	558 (94.7)		56 (9.5)	
		Yes	31 (5.3)		533 (90.5)	
	**Extrinsic motivation in clinical learning,** **n (%)**					
		No	506 (85.9)		106 (18.0)	
		Yes	83 (14.1)		482 (81.8)	
	**Amotivation,** **n (%)**					
		Yes	475 (80.6)		313 (53.1)	
		No	114 (19.4)		276 (46.9)	

^a^Field data (2021).

### Overall Mean Score Change and Mean Difference in Motivation in Clinical Learning Between Pretest and Posttest Among Nursing Students

As shown in Table 5, there was a statistically significant increase in mean scores changes of motivation in clinical learning from mean 9.31 (SD 2.315) at baseline to mean 20.87 (SD 5.504) at the end line. A comparative analysis of motivation performance among nursing students between pretest and posttest was found to be statistically significant (mean 11.566, SD 5.667; t_588_=49.496; *P*<.001; 95% CI 11.107-12.025). The findings suggest that nursing students scored high on motivation in clinical learning in the posttest as compared with the pretest. Moreover, the findings in Table 5 indicated that there was an increase in mean scores among nursing students per domain of motivation to clinical learning between the pretest (mean 3.74, SD 1.231) and the posttest (mean 9.53, SD 2.762).

**Table 5:** Overall mean score change and mean difference in motivation in clinical learning between pretest and posttest among nursing students (N=589)^a^.

		Pretest, mean (SD)		Posttest, mean (SD)		Mean difference, mean (SD)		*t* test (*df*)		*P* value		95% CI		Effect size (Cohen *d*)		95% CI	
Motivation in clinical learning			9.31 (2.315)		20.87 (5.504)		11.56 (5.667)		49.496 (588)		.001		11.107-12.025		2.743		1.011-4.107
**Domains of motivation in clinical learning**																	
	Intrinsic		3.74 (1.231)	9.53 (2.762)		5.800 (2.968)		47.421 (588)		.001		5.559-6.040		N/A^b^		N/A	
	Extrinsic		4.49 (1.42)	8.77 (3.325)		4.276 (3.474)		29.845 (588)		.001		3.994-4.557		N/A		N/A	
	Amotivation		2.57 (1.1871)	1.08 (1.392)		1.492 (1.644)		22.031 (588)		.001		1.359-1.625		N/A		N/A	

^a^From field data (2021).

^b^N/A: not applicable.

Moreover, they also demonstrate higher scores in their extrinsic motivation to learning in clinical settings at the end line (mean 8.77, SD 3.325) than at baseline (mean 4.49, SD 1.42) while amotivation performance decreased from mean 2.57 (SD 1.187) at baseline to mean 1.08 (SD 1.392) at the end line. The effect size of the intervention on motivation in clinical learning among nursing students was computed using Cohen *d* formula (mean 2 minus mean 1 divided by a pooled SD). Findings showed that the intervention demonstrated an effect size of 2.74 (*P*<.001; 95% CI 1.011-4.107), which is a high effect size based on Cohens *d* classifications of effect sizes [46].

### The Estimated Effect of an Intervention (Interactive Web-Based Clinical Practice Monitoring System) Controlled for Other Co-Related Factors on Motivation in Clinical Learning Among Nursing Students at Posttest

About 58.5% variation in motivation in clinical learning scores is explained by the explanatory variables included in the model. The overall model was statistically significant (*f*=25.6; *P*=.001). Findings in Table 6 indicate that reasons to join the nursing profession such as due to the opportunity to demonstrate autonomy (β=1.590; *P*=.02; 95% CI 0.279-3.901), the opportunity to travel around the world (β=1.648; *P*=.04; 95% CI 0.583-4.713), job availability (β=1.409; *P*=.001; 95% CI 1.046-5.772), and other corelated factors were statistically significantly associated with motivation in clinical learning among nursing students against their counterparts. The estimated effect (β) of a 3-week intervention to improve nursing students’ motivation in clinical learning was 3.041 (*P*=.03, 95% CI 1.022-7.732) when controlled for other correlated factors.

**Table 6:** The estimated effect of an intervention (interactive web-based clinical practice monitoring system) controlled for other corelated factors on motivation in clinical learning among nursing students at posttest (N=589)^a,b^.

Variable				Estimate (β)		SE		*P* value		95% CI
**Intervention**										
	Pretest		1		N/A^c^		N/A		N/A	
	Posttest		3.041		0.308		.03^d^		1.022-7.732	
**Institutions**				N/A		N/A		N/A		N/A
	Institution A		1		N/A		N/A		N/A	
	Institution B		.729		0.883		.23		1.956-0.467	
	Institution C		.932		0.794		.65		6.052-3.753	
	Institution D		.312		1.117		.12		0.194-1.757	
**Programs**										
	Diploma		1		N/A		N/A		N/A	
	Bachelor		.281		0.483		.56		1.229-0.668	
**Year of study**										
	Second-year diploma		1		N/A		N/A		N/A	
	Third-year diploma		.593		0.809		.36		1.806-0.655	
	Third-year bachelor		.936		0.926		.71		5.315-3.611	
	Fourth-year bachelor		.744		0.617		.01^d^		0.431-3.417	
**Age group (years)**										
	<24		1		N/A		N/A		N/A	
	24-34		1.149		2.496		.49		0.669-1.407	
	>35		.782		0.497		.42		1.827-0.768	
**Sex**										
	Female		1		N/A		N/A		N/A	
	Male		.434		0.905		.58		0.851-1.512	
**Marital status**										
	Single		1		N/A		N/A		N/A	
	Married		.575		0.627		.39		1.184-3.007	
**Accommodation**										
	In-campus		1		N/A		N/A		N/A	
	Off-campus		.852		2.272		.73		1.981-1.385	
**Interested to join the nursing profession**										
	No		1		N/A		N/A		N/A	
	Yes		.250		0.043		.051^d^		0.335-0.165	
**Reason to join nursing**										
	**Autonomy to practice**									
		Yes	1.590		0.667		.02^d^		0.279-3.901	
		No	1		N/A		N/A		N/A	
	**Caring patients**									
		Yes	1.107		0.679		.10		0.226-2.441	
		No	1		N/A		N/A		N/A	
	**Opportunity to travel around worldwide**									
		Yes	1.648		1.512		.04^d^		0.583-4.713	
		No	1		N/A		N/A		N/A	
	**It is the secured profession**									
		Yes	.563		0.551		.31		0.519-1.645	
		No	1		N/A		N/A		N/A	
	**Reasonable payment**									
		Yes	.033		0.538		.95		1.089-1.023	
		No	1		N/A		N/A		N/A	
	**Job availability**									
		Yes	1.409		0.694		.001^d^		1.046-5.772	
		No	1		N/A		N/A		N/A	

^a^From field data (2021).

^b^*R*^2^=0.865, *f*=116; *P*<.001, significant at *P*<.05, and significant at *P*<.001.

^c^N/A: not applicable.

^d^Variables that are significantly associated with the outcome variable.

## Discussion

### Principal Findings

In terms of the study’s focus and objective, the implementation of an interactive web-based clinical practice monitoring system for nursing students’ motivation in clinical learning was feasible and practical in a clinical setting with consistent electricity supply and web connectivity. Nursing students indicated moderate to high levels of motivation in clinical learning after 3 weeks of system implementation, compared with when they were not exposed to it. Nursing students demonstrated a capacity to plan, identify, and access academic resources and help, as well as participate in clinical practices with minimal support from clinical instructors, trainers, and academic faculty, according to the end line assessment. Nonetheless, contrary to the existing practices where nursing students are not allowed formally to use electronic devices in clinical settings, the use of electronic devices while students are in clinical settings such as smartphones, iPads, and computers would most likely extrinsically motivate nursing students to attend their daily duty shifts.

The posttest results show that nursing students who are mentored, supported, monitored, supervised, and evaluated using an interactive web-based clinical practice monitoring system are more efficient in terms of clinical attendance and completing clinical activities on time. The findings of this study indicated that nursing students showed a readiness to stick to their clinical duty roster, report on the clinical environment on time, receive and give reports, and complete their assigned responsibilities using an interactive web-based clinical practice monitoring system. Furthermore, students expressed a desire to ask questions and locate clinical resources to help them learn not just to win a prize or a grade but also to broaden their knowledge and skills. Despite a significant change in clinical learning motivation among nursing students, the system improved clinical attendance as an indicator that it motivated and enhanced their interest and willingness with a sense of being confident, independent, and autonomous to engage in clinical learning activities than when conventional clinical pedagogies such as attendance books, bed tutorials, or assignments were predominantly used.

Similarly to the findings from other previous scholars [2,21], the implementation of an interactive web-based clinical practice monitoring system would allow nursing students to interact with one another while performing clinical nursing procedures, as well as interact instantly and timely with trainers, clinical instructors, or academic faculty for any support or mentorship. Nonetheless, as argued by some previous scholars [5,47] that individuals’ behaviors are sometimes shaped by their personalities, this study found that nursing students’ motivation in clinical learning was partly attributed to what motivated them to join nursing education programs, such as challenges in job availability, opportunities to demonstrate autonomy in nursing, and opportunities to travel around the world when individuals enrolled in the nursing profession. Such corelated aspects to the intervention would most likely drive nursing students to study nursing programs extrinsically rather than intrinsically to match their academic and living goals.

Referring to the findings from some previous scholarly works on students’ abilities to demonstrate an interest in their learning activities becomes a precursor for them to be motivated to identify and locate learning resources and thus engage in learning activities actively [48,49]. Similarly, scholars’ [6,50,51] low motivation in clinical learning has been linked to uninteresting clinical teaching techniques, an uncomfortable learning environment, a lack of interest, and unclear clinical objectives, all of which contribute to clinical absenteeism among nursing students. This study’s findings on motivation to learn are consistent with those found by Millanzi and Kibusi [6], for example, who argued that innovative pedagogy has the potential of improving learning motivation among nursing students.

Nevertheless, Allvin et al [52], claimed that while the clinical environment is important for nursing students’ academic achievement, students’ learning motivation is positively correlated with an adequate number of competent and qualified clinical instructors who use innovative clinical pedagogies that enhance their motivation in clinical learning, including the prescription and integration of interactive web-based clinical practice monitoring system in nursing curricula. In the same way, Aghajari et al [53] observed that the majority of nursing students lack academic motivation during clinical placement practices because the clinical learning environment, as well as clinical teaching and learning pedagogies, does not motivate them to learn and meet their clinical academic potential as lifelong learners. This study’s and prior research findings indicate to underline that a favorable, learner-centered, and technology-based innovative clinical pedagogy may positively boost clinical learning motivation among nursing students in nursing education.

### Limitations of the Study

The study did not involve a control group to maximize the validity of findings on the efficacy of interactive web-based clinical practice monitoring systems on the outcomes of interest. The use of a single group may obscure the interpretation of the effect size on the outcome variables because standard clinical teaching pedagogies would also produce effects on the outcome of interest. Therefore, findings on the effect of interactive web-based clinical practice monitoring systems need to be interpreted cautiously by considering this limitation. The study suffers from a methodological limitation, as it did not adopt a randomized controlled trial (a true intervention) to estimate the random effect of the interactive web-based clinical practice monitoring system on the outcome variables over the standard clinical teaching pedagogies. Therefore, findings need to be treated and interpreted cautiously by considering that with a quasi-experimental design random effect of the intervention on the outcome, variables would not be established without outweighing its effect over the standard clinical teaching pedagogy (control group) if it could be involved.

### Conclusions

The findings of this study demonstrate that it is possible to teach, mentor, supervise, support, monitor, and assess nursing students throughout their clinical placements by adopting, implementing, and evaluating an interactive web-based clinical practice monitoring system. As a minimum exposure of at least 3 weeks of the interactive web-based clinical practice monitoring system, nursing students may demonstrate nursing students’ attendance and motivation in clinical learning by their will than is now performed, where sanctions and other associated techniques are used to force them to attend their daily clinical duty shifts accordingly. Incorporating technology into clinical nursing education pedagogics nursing curricula can be an alternative educational technique for educators in nursing education to facilitate clinical learning activities to develop motivated and passionate undergraduate nursing students to engage and learn efficiently and effectively during their clinical placements. Nonetheless, managing a big group of nursing students was proven to be achievable with the use of an interactive web-based clinical practice monitoring system.

Furthermore, an interactive web-based clinical practice monitoring system was feasible to not only mentor, supervise, monitor, and support nursing students but also record students’ clinical attendance and the type and number of clinical nursing procedures learned and practiced, as well as generate clinical formative evaluation reports. In other words, an interactive web-based clinical practice monitoring system can be used as an innovative clinical pedagogical approach in clinical teaching and learning to improve nursing students' motivation in clinical learning as a precursor to clinical competence for quality and cost-effective care to people.

## Data Availability

The datasets generated during and/or analyzed during this study are available from the corresponding author on reasonable request via patriciaz1006@gmail.com.
